# Unraveling the polychromy and antiquity of the Pachacamac Idol, Pacific coast, Peru

**DOI:** 10.1371/journal.pone.0226244

**Published:** 2020-01-15

**Authors:** Marcela Sepúlveda, Denise Pozzi-Escot, Rommel Angeles Falcón, Nicolas Bermeo, Matthieu Lebon, Christophe Moulhérat, Philippe Sarrazin, Philippe Walter

**Affiliations:** 1 Universidad de Tarapacá, Instituto de Alta Investigación, Arica, Chile; 2 Sorbonne Université, Laboratoire d’Archéologie Moléculaire et Structurale (LAMS), CNRS UMR, Paris, France; 3 Archéologie des Amériques (ArchAm), CNRS- Université Paris 1 Panthéon Sorbonne UMR, Paris, France; 4 Museo de sitio Pachacamac, Lurín, Lima, Perú; 5 Université Paris 1 Panthéon-Sorbonne, Paris, France; 6 Muséum National d’Histoire Naturelle, Histoire Naturelle de l’Homme Préhistorique, CNRS UMR, Paris, France; 7 Département du Patrimoine et des Collections, Musée du Quai Branly Jacques Chirac, Paris, France; 8 SETI Institute, Mountain View, California, United States of America; Universita degli Studi di Milano, ITALY

## Abstract

Pachacamac is the name of the 15th-16th century Inca sanctuary on the Peruvian coast as well as the name of one of the principal oracles of Inca divinities. This effigy would have been destroyed by Pizarro in 1533 during his visit to the great monumental complex, and as such the originality and antiquity of the wooden statue—the so-called Pachacamac Idol—have been the subject of much controversy and debate. We present here previously unpublished dates that confirm its manufacture during the Middle Horizon (AD 500–1000), as well as evidence of its original polychromy. Traces of colors were observed on its different sections with portable microscopy and analyses with two different X-Ray Fluorescence spectrometry techniques, leading to identification of yellow, white, and red mineral pigments, including the presence of cinnabar. Dated between the 8th and 9th centuries, the statue would have been worshipped for almost 700 years, from the time of its creation to the time of the Spanish conquest, when Pachacamac was a major place of pilgrimage. These data not only offer a new perspective on Pachacamac’s emblematic sacred icon, but also on the colorful practices of the Pre-Hispanic Andes.

## Introduction

The use of colored paint produced from mineral pigments on the surfaces of sculptures and architecture relating to the religious cults of ancient Greece and Rome has been widely demonstrated. For many centuries these material expressions were thought to have been monochromatic—given the lack of empirical evidence confirming their polychromy; a paradigmatic illustration of a past idealized as pure and white [1,2]. These works are now seen in their original polychrome as the result of varied chemical analyses [3,4]. In a similar fashion, the Pre-Hispanic Americas were not exempt from the use of colorants and colored materials, as is demonstrated, for example, in the extensive mural painting tradition in Mexico [5–8] and Peru [9–11], and the rock art of different regions of South America [12]. The minerals found ground or whole, the painted objects–ritual offerings–found in many funerary and ceremonial contexts [13,14], and the identification of sites where mineral pigments were extracted in different regions of the Andes (dating from the late Pleistocene to the 16th century) also demonstrate their importance [15–17]. Despite several references to the use of color and the different associated color terms collected by chroniclers since the 16^th^ century (see multiple references in [18]), there is little knowledge of the specific symbolic value and meaning of colors in the Pre-Hispanic Andes. In addition to problems with conservation and the lack of analyses, the polychromy of objects and places of worship is poorly understood because many temples, idols, and icons were destroyed, some in Pre-Hispanic times, but many more since the Spanish conquest [19]. Nevertheless, the importance of color is clearly demonstrated in archaeological studies. For example, investigations relating to Andean metallurgy illustrate that the most important property in metals was thought to be the color of gold, observed on the surface of objects and ornaments, and in this region always associated with political power, social status and religious belief. Gold was the color related to the divinity of the sun, whereas silver was associated with the moon [20,21]. To this end, ancient metallurgists developed specific technologies and different alloys, but the treatment applied was always directed towards obtaining a gold color on the surface of the metal objects. Colors on textiles (dyed or painted) and their structure and arrangement on clothing and head-dresses was highly significant and contributed to the identification of individuals based on their status and privileges, but also of different Andean social communities [22–24]. Nevertheless, despite this additional evidence, there is little information on the materiality of Pre-Hispanic mineral pigments and paintings, with reference to its material (composition) and immaterial (knowledge and symbolic dimensions), that is related to its provenance (direct or through exchange), production and consumption [13,14,25–27]. Given this, we began a broad study of the vestiges of polychromy in the Pachacamac archaeological complex, examining painted murals and colored objects, as well as artifacts related to color production or application (e.g., brushes and textiles).

**Fig 1:**
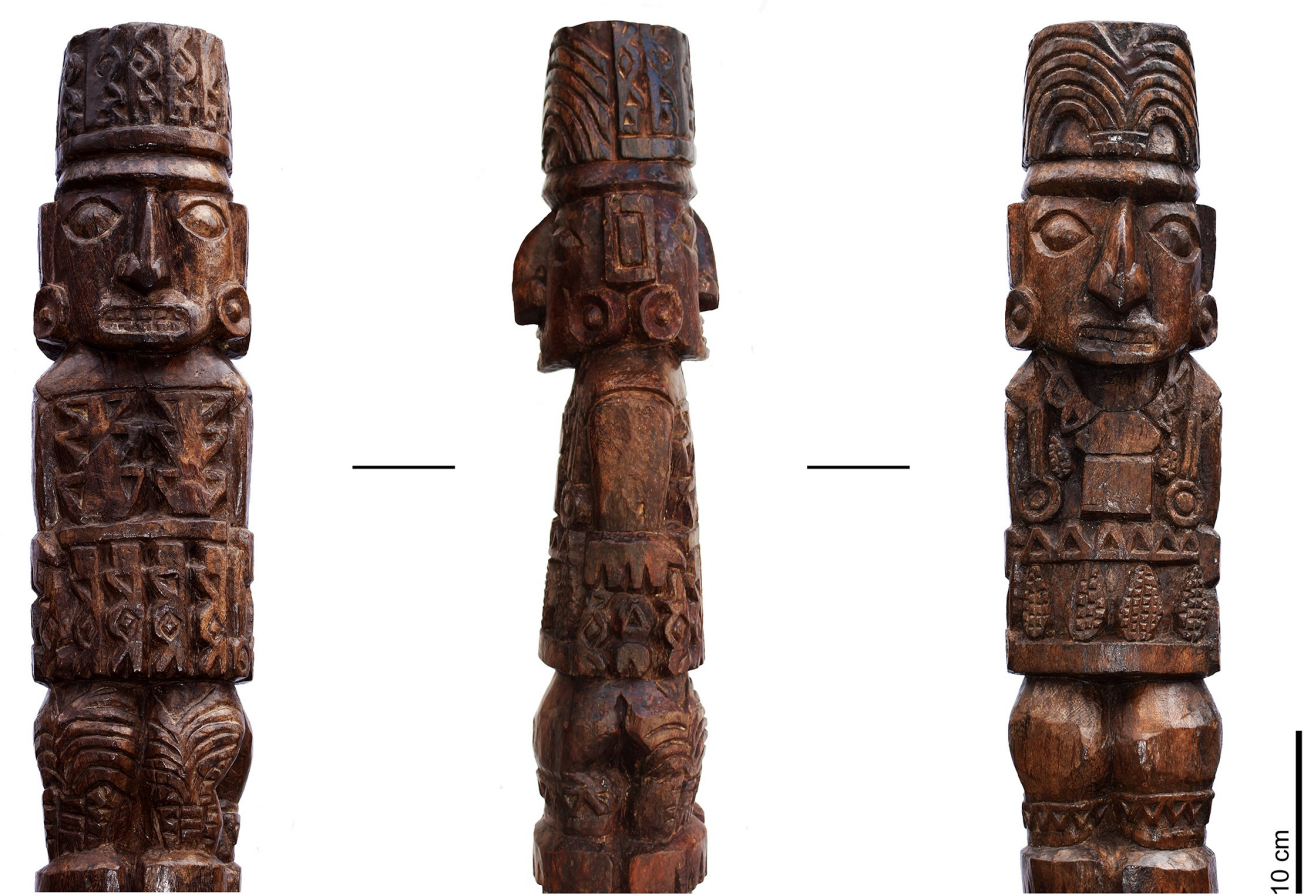
The wooden statue of the Pachacamac Idol.

This work presents results obtained from an original *in-situ* non-destructive analysis using two types of X-Ray Fluorescence spectrometry—mapping XRF employed for first time on South American cultural heritage—of traces of colors observed in different locations on the venerated statue, the so-called Pachacamac Idol. These findings help to demonstrate the original polychromy of this sacred wooden statue, which has not been studied to date (Fig 1). We discuss the implications of this identification and the first radiocarbon dating of the wood of the Idol, which is still part of a religious cult in one of the most important pilgrimage sites of the Pre-Hispanic Andes.

## Material and methods

### The Pachacamac Idol

The Spanish conquest of the South American Andes caused tremendous upheaval among local populations, changing their ways of life, belief systems, social and political organization, and economies [28,29]. Even prior to the institutionalized Extirpation of Idolatry in the 17th century, early post-contact accounts by colonists attest to violent acts against *wak’as*, idols, and worshipped objects of a variety of materials, including wood, stone, metal, painted on walls and rock surfaces, as well as against sacred places [19,30,31:154]. It was in this context that the Pachacamac Idol, venerated at the monumental complex bearing its name, was reportedly destroyed. Associating it with the image of the devil, in 1533 Hernando Pizarro ordered his followers to, “*undo the vault where the idol was and break him in front of everyone*” [32:133] (S1 Text).

Historic sources report that this icon, considered to possess oracular properties, was one of the most important deities of Pre-Hispanic times, and one of the principal Inca oracles in the 15th and 16th centuries [33–35]. After being consulted by the *Inca Tupac Yupanqui*, who was known for having introduced sweeping reforms in the large territory conquered by the Inca, the site of Pachacamac became part of a complex network of settlements and roads in the *Tawantinsuyu* empire. Through the construction of monumental edifices, modification of existing structures and the practice of both human and animal sacrifice at the site as a strategy of symbolic and political expansion, *Tupac Yupanqui* definitively sealed the *Incas’* possession and control of Pachacamac, and established a new order that enhanced the importance of this major sanctuary [36–38].

**Fig 2:**
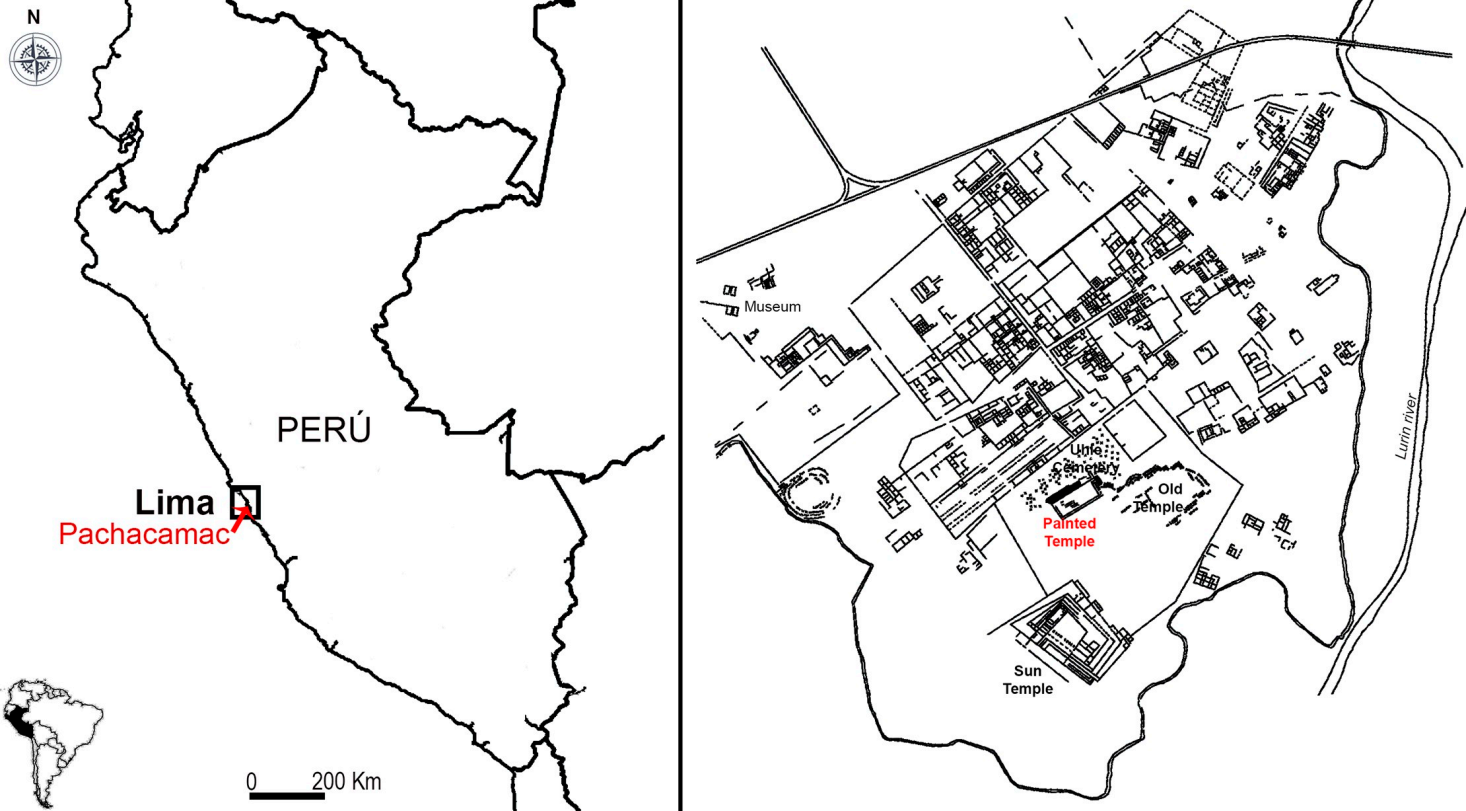
Map of Pachacamac archaeological complex and the location of the principal temples.

The Pachacamac complex, still visible today at the mouth of the Lurín Valley, 31 km south of the present-day city of Lima in Peru (Fig 2), bears testimony to a long occupational history that began in the early centuries before the present era, around 200 BC [39–41]. Many of the earliest structures can be found beneath the major remodeling carried out later by the *Inca*, and before them by the *Lima*, *Huari* or *Wari*, and *Ychsma* people. The monumental architectural complex of Pachacamac covers nearly 450 hectares and consists of many buildings used as palaces and temples, as well as squares, and roads to enable transportation. Three walls divided the site into different sectors. Outside the third—and outermost—wall, residential districts and handicraft workshops are evidence of the everyday activities that sustained life in Pachacamac, and yet were carried out on the margins, set apart from the acts of worship that took place in the site’s inner sanctum. This private area, enclosed inside the first wall and situated at the highest point of the site, includes three great structures: the Old Temple, the Painted temple, and the Temple of the Sun, constructed with adobe and carved rocks, as well as a large cemetery assigned to the Middle Horizon (AD 500–1000) and consisting of individual graves whose occupants were buried with rich offerings [39–41]. Both the Temple of the Sun and the Painted Temple still conserve extensive areas with vestiges of murals decorated with human, marine, and geometric motifs painted in red, yellow, black, green, and white [5,42–44].

Upon these structures with restricted access, dark vaults were built to house idols and divinities that were cared for by priests and high-ranking officials. In 1534, De Xerez notes “*The idol was in a good well painted house*, *in a very dark*, *smelly and very closed room; they have an idol made of very dirty wood that they say is their god who raises and sustains them and increases their wealth*.*” “It was found that the devil is inside the Idol*, *and speaks with those who are his allies* […] *They regard him as god*, *and make many sacrifices to him*" [32:131] (S2 Text). The Idol was also described by De Estete between 1530 and 1533: *"* […] *a very small cave*, *rough*, *without any work; and in the middle of it was a tree*, *driven into the ground*, *with a man's figure made on his head*, *poorly carved and malformed* …*”* [45:262] (S3 Text).

It was in 1938—on the occasion of the VIII Panamerican Conference—that Albert Giesecke excavated the “Atrio central”, the upper part of the Painted Temple (Fig 2), and discovered a large and cylindrical carved wooden post, more than 2.34 meters tall with an approximate diameter of 13 cm, and decorated almost entirely with carved figurative motifs [43]. This structure had previously been partially excavated by Uhle when the Peruvian government decided to exhibit certain elements of these monumental structures to international visitors [41]. More recently, the Painted Temple was excavated by Shimada and collaborators from the Pachacamac Archaeological Project [40], and the archaeological unit of the Museo de Sitio de Pachacamac [44]. Discovered at a similar place as the painted building referred to by the chroniclers, the wooden statue was named the Pachacamac Idol.

**Fig 3:**
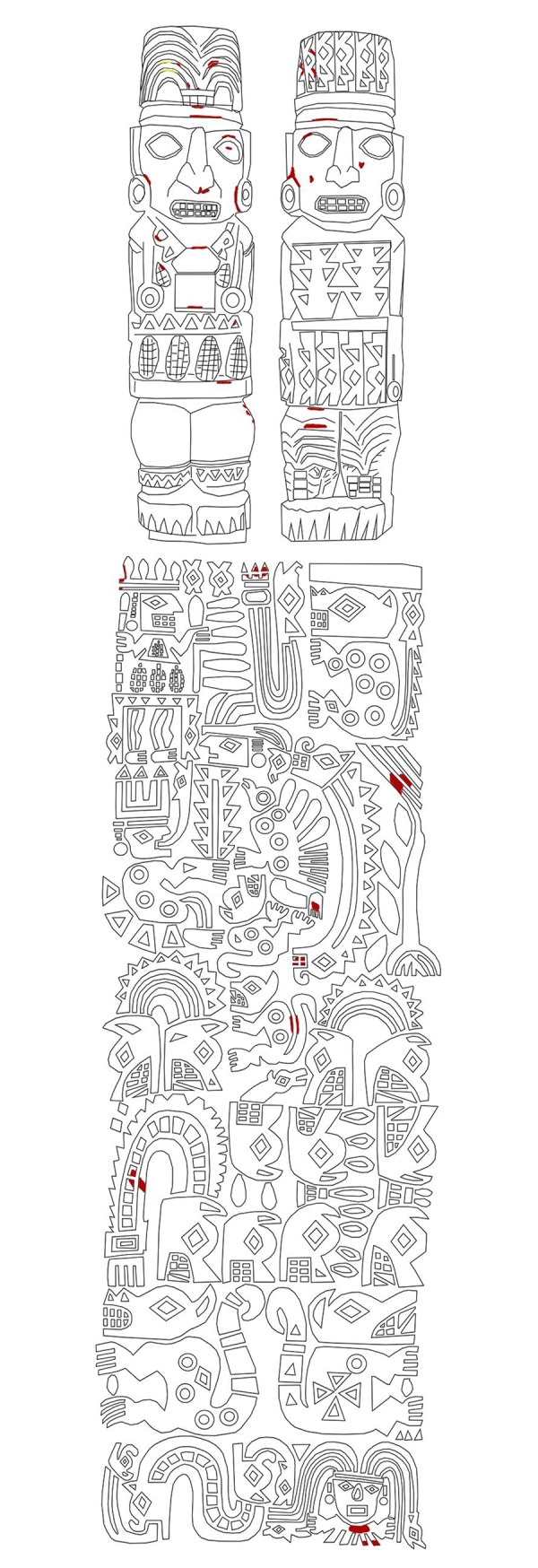
Traces of paint on the Pachacamac Idol (except white, which is indicated in grey, the others -red and yellow- are represented by their own colors).

The post displays three clearly identifiable sections (Fig 3). The upper part consists of two front-facing human figures standing next to each other without any apparent association between them. Each wears a distinctive head-dress and attire, and on both there are traces of color. The first—called “Personaje A”—wears a feather headdress with vestiges of red and yellow, and evidence of red on his face and body ornaments; the second—“Personaje B”—wears a snake headdress with traces of red, and of red and white on his face. The middle and largest segment of the post bears multiple, richly attired human figures, animals with human heads, other animals (felines with mottled hides and two-headed snakes) and geometric motifs, with traces of red and yellow. The third and lowest segment is not decorated and may have been the part inserted into a pedestal (Fig 3).

Iconographic and stylistic analyses of the decorations carved on the Idol, including a comparison with characteristic motifs and wooden artifacts of the Wari culture, allowed a preliminary assignment of the post to the Middle Horizon [33–35,43], however other authors assigned it to the Late Intermediate or *Inca* periods [46], interpretations that remained unconfirmed until our analyses. Other studies explored the possible significance of the icon with a different approach, relying primarily on Colonial-era chronicles and documents [33–35,47–51]. When the *Incas* arrived, Pachacamac was transformed into one of the leading places of pilgrimage of the *Tawantinsuyu* empire, attracting devotees from across the Andes who came to consult the Idol turned oracle, and leaving offerings of gold, silver, and garments, among other items [51].

## Methods

A sample of wood from the Pachacamac Idol was obtained from a natural hole on the third and lowest part of the artifact (S1 Fig). Before being dated, the wood sample was cross-sectioned to aid in its classification. Taxonomic identification of wood based on cellular tissue anatomy relies on the observation and characterization of different cellular types (fibers, parenchyma, vessel member, etc.) in the secondary xylem. These can include size, quantity, arrangement, aperture types, and cellular wall properties, among other elements [52]. Observations were conducted on three anatomical planes or sections: transverse, longitudinal tangential, and longitudinal radial, to allow each element to be examined in its entirety, as some characteristics can only be appreciated in specific planes [53,54]. Taxa may be differentiated based on the specific combination of various criteria or features displayed by specific elements of the cellular tissue [53,54]. This may enable identification up to the level of species or variety for taxa displaying peculiar and distinctive elements, if these have been described in detail. Indeed, in order to recognize a particular taxon based on its anatomical characteristics, these must have been observed and documented beforehand from specimens correctly identified by other methods. To make the most accurate identification, one would ideally have access to comparative samples. Wood samples are usually prepared by obtaining a thin section that can be mounted for viewing under an optical microscope [54]. The nature of the sample discussed here prevents this approach. Instead, the sample was observed with a reflected light microscope, as is often done with charcoal fragments. As external surfaces of the samples are often worn and do not display any distinguishable cellular elements, the sample was further divided to obtain clear observation planes as described above.

**Fig 4:**
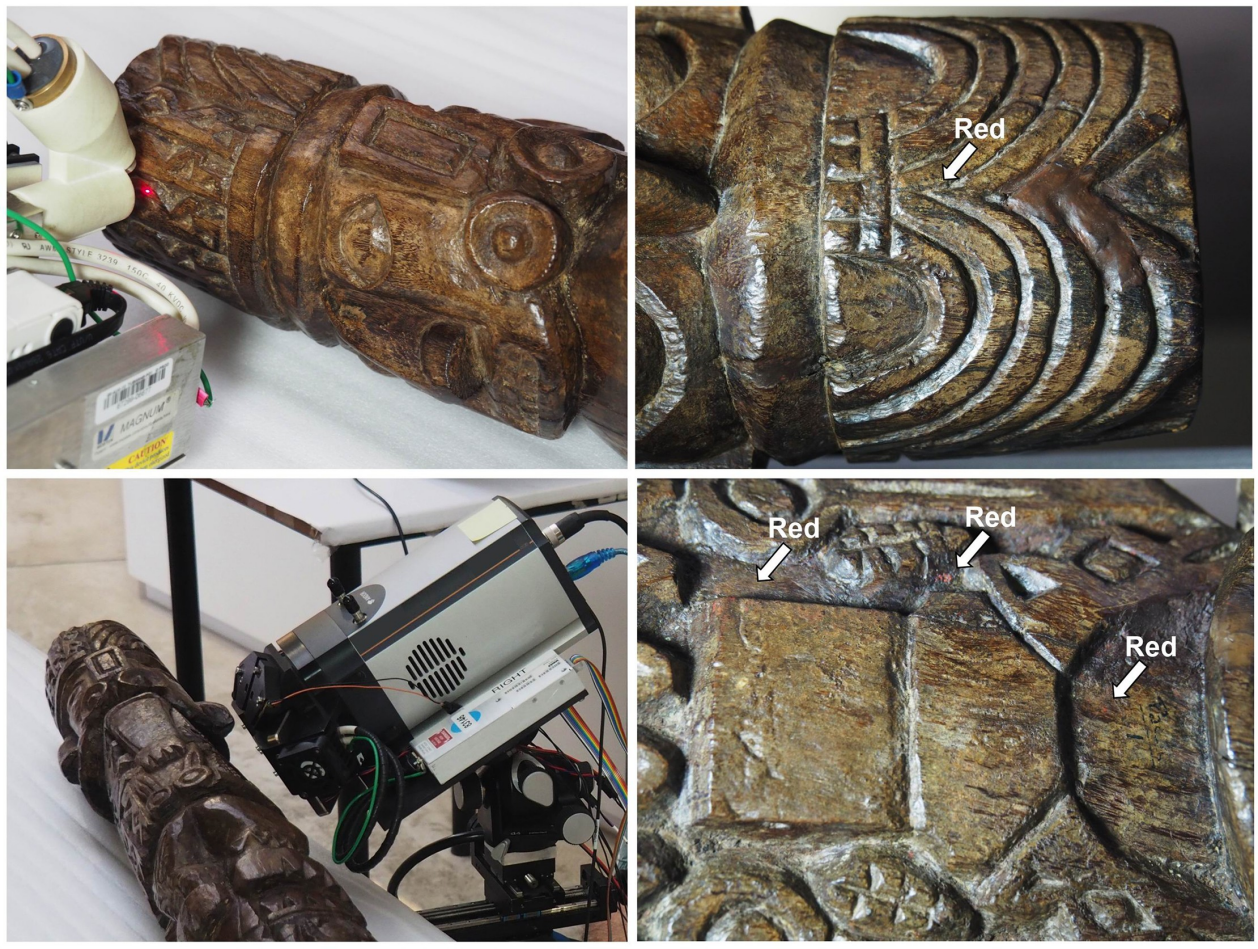
*In-situ* analysis of the Pachacamac Idol.

The remains of pigments were observed under microphotography (Olympus OM-D E-M5 Mark II with an Olympus 80mm macro lens) and with a digital microscope (Dino-lite, AnMo Electronics Corporation, New Taipei City, Taiwan). An in-house-built XRF instrument was used for the analysis, featuring a Pd anode end window X-ray tube (Moxtek MAGNUM, Orem, UT) operated at 30 kV and 50 μA and a silicon drift detector (X-123FAST SDD, Amptek, Bedford, MA) with an active area of 70 mm^2^ collimated to 50 mm^2^ and a nominal thickness of 500 μm (Fig 4). The X-ray tube was connected to the detector via a holder produced by 3D printing (fixing the angle between both to 45° for single point analysis, or 32° for imaging). As a collimator primary optic, a Pd tube of 800 μm inner diameter was used, yielding a beam size of approximately 1.2 mm. The typical working distance is 1 cm. The system was used with manual translation stages for single-point analysis with an acquisition time of 300 s.

XRF mapping was carried out with the Mapping X-ray Fluorescence Spectrometer (“Map-X”) that provides elemental imaging at ~100μm spatial resolution (refMap-X;55). Map-X is placed at 25mm from the surface of an object and held in a fixed position during the analysis (Fig 4). A 13x13 mm^2^ area of the surface is uniformly illuminated with X-rays produced by two Au anode X-ray tubes (Moxtek, MAGNUM, Orem, UT) and in turn emits X-rays by fluorescence. A Micro Pore Optic (PHOTONIS), often referred to as “lobster eye optic,” collects a fraction of this X-ray emission and focuses it on a CCD detector operated at up to a few frames per second (Andor iKon M operated at -60°C). Single photon processing allows the measurement of energy and coordinates of each X-ray photon collected. A large set of frames is reduced into a 2d histogram processed into higher level data products such as elemental maps for elements of interest [55]. All the XRF spectra were processed with PyMCA software [56].

## Results

The sample wood from the Pachacamac Idol is composed of fragments of mature as well as juvenile wood, which is characterized by its notable ring curvature and the presence of pith cells. Anatomical elements were more visible in some fragments than others but were always present (S2 Fig). The wood is heteroxylous and diffuse-porous, with a distinct growth ring boundary marked by marginal parenchyma containing prismatic crystals. Vessels are almost exclusively solitary but can also appear in multiples of two; they present a circular outline and do not appear to be arranged in any discernible pattern. In the juvenile fragment, vessels have an average density of 20/mm^2^ and an average of 36 μm in tangential diameter (range 15–71). In the mature wood fragments only two vessels were visible in transverse view with tangential diameters of 105 μm and 66 μm. Vessel perforation plates are simple and inter-vessel pits are alternate, oval to angular and vestured, measuring between 4 and 7 μm. Vessel-ray pits are similar to inter-vessel pits. No tylose or spiral thickening are visible. Axial parenchyma is vasicentric, sometimes confluent and appears in at least one marginal band in the juvenile wood. Parenchyma appear as single fusiform cells and two cells per strand, but three cells per strand are also found. Prismatic crystals are present in chambered parenchyma cells. Rays are uniseriate and 2-(3) seriate, and 5–20 cells high in juvenile wood and multiseriate (2–4 cells wide) and up to 30 cells high in mature wood. Rays are homocellular with all procumbent cells, and number 5–8 per millimeter. Fibers are non-septate and thin walled (2 μm) (S2 Fig). The wood is undoubtedly from the Leguminosae (Fabaceae) family, and very probably from the Mimosoideae subfamily, possibly either Prosopis sp. (*P*. *pallida* or *P*. *juliflora*) or *Vachellia macracantha*. The radiocarbon date obtained is 1289 +/-25 BP (cal. 760–876 AD; D-AMS 028819), corresponding to the Middle Horizon (S1 Table).

**Fig 5:**
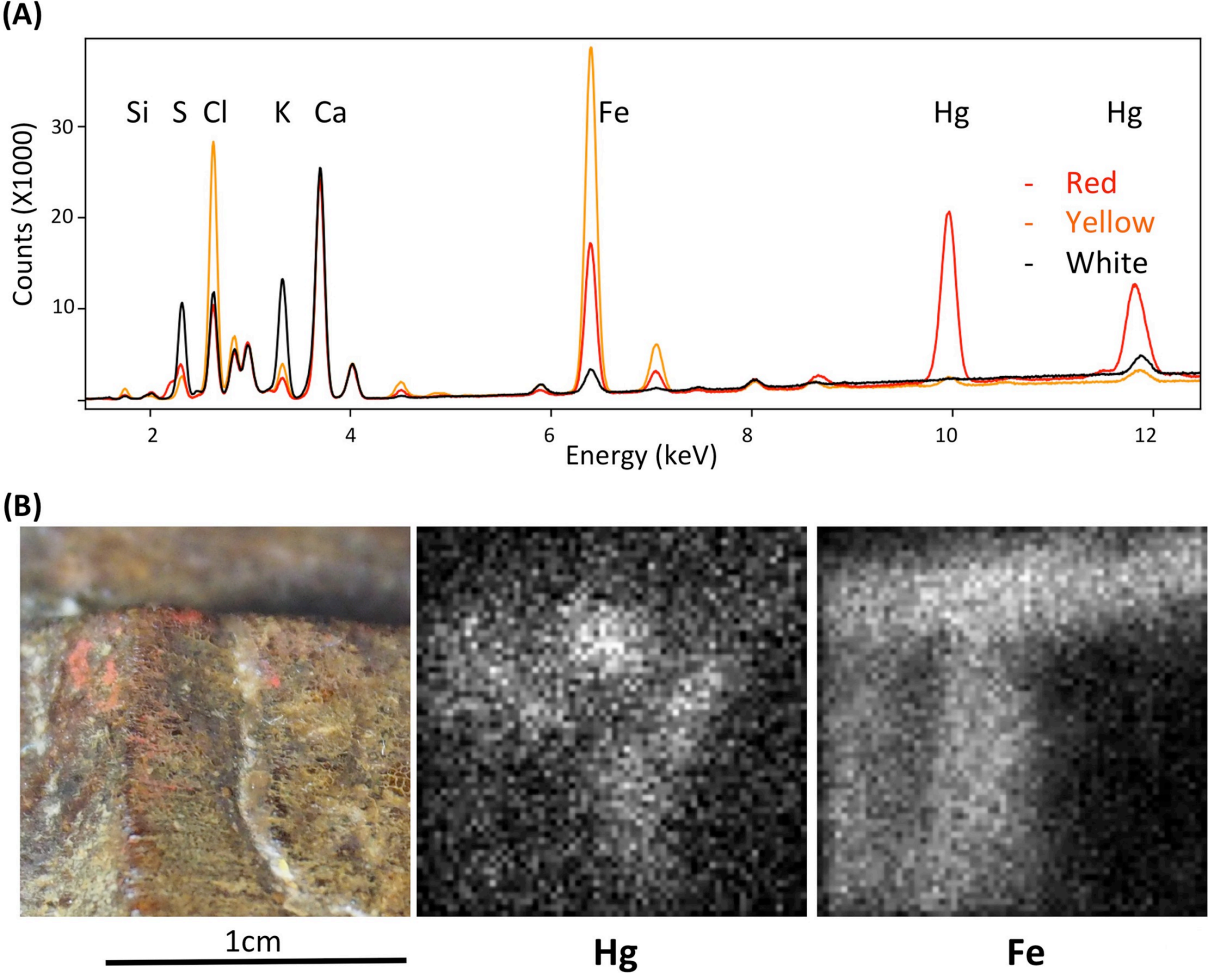
5a. X-ray fluorescence spectra of the 3 analysed colored traces; 5b. X-ray fluorescence mapping of the red color.

Polychromy was analyzed using two different types of X-ray fluorescence (XRF) spectrometry. The traces of color are very localized in the upper and middle sections, and the entire sculpture shows an accumulation of dirt in crevasses and recesses and a thick layer of varnish that absorbs low energy X-rays (Fig 5A). Precise identification of the paint would have required the collection of samples, which the authors elected against in order to preserve the traces that remain on this unique object and to contribute to pre-Hispanic cultural heritage conservation. We identified three color residues. The red pigment is clearly composed of cinnabar, a red mercury sulfide (HgS) [57,58], as confirmed by X-ray fluorescence mapping (Fig 5B) that shows the correlation between the red spots and the Hg signal. The yellow color contains mainly iron and is likely to be an earth pigment or iron oxide such as goethite FeOOH or jarosite K^+^Fe^3+^_3_(OH^−^)_6_(SO_4_^2−^)_2_. In the white color, potassium and sulfur were observed, and given the calcium content in all measurements, gypsum is assumed to be the main pigment and the higher potassium content is a result of contact with the air or the adobe materials. Chlorine is also present in various concentrations in all measurements, which is consistent with the sculpture's exposure to the atmosphere, rich in sodium chloride due to the proximity to the ocean. We cannot exclude other types of mineral for the white pigment.

## Discussion

Based on the anatomical observation of the sample, the wood of the Pachacamac Idol is definitely not *lúcumo* (*Pouteria lucuma*), from the family Sapotaceae, as previously thought [34,43,59]. This identification also differs from the results of another earlier taxonomic analysis of three other wooden statues from Pachacamac, acquired by Captain Berthón in 1911 and offered to the Musée du Trocadero in Paris, today belonging to Musée du Quai Branly Jacques Chirac. These others were effectively recognized as *lúcumo* [59]. However, the Leguminosae (Fabaceae) family of the wood of the Pachacamac Idol—either Prosopis sp. (P. pallida or P. juliflora) or Vachellia macracantha–corresponds to a species previously recognized in anthracological investigations of wood charcoal assemblages in different areas of the site where it was used use as fuel [59,60]. The tree used to make this post was very probably obtained in the vicinity of an agricultural area surrounding the monumental pilgrimage center. De Xerez described the area as being densely forested when he arrived at the Lurin valley with Pizarro [32]. The wood of the effigy was probably chosen for its hardness and resistance, but also for its size and dark color. We cannot of course exclude a special symbolic value of this species of tree, as was established for the *lúcumo* in pre-Hispanic times [59].

The pigments identified on the wooden post coincide with those previously analyzed in different regions of the Central Andes on offerings and mural paintings since at least the Formative period, centuries before the beginning of the present era [9,11,18,61–67]. While the yellow and white paints used for the murals of the Painted Temple were produced in Pachacamac from mineral pigments available around the site [42,44], the occurrence of cinnabar in Andean geology is rare and none has been found around the Pachacamac site [68], so it is very unlikely that its presence on the buried Idol has occurred naturally. This therefore constitutes proof that the object was deliberately painted, with at least three colors. At Pachacamac, cinnabar was only previously found in powdered form in textile pouches deposited as offerings or applied on other supports such as wooden masks in funeral bundles [69].

The use of vermilion, a pigment obtained from the powdered cinnabar mineral, is widely documented in the Andes since before the Formative period (1500 BC-500 AD) as an offering and as a pigment mixed with iron oxides [14]. Later, from the Middle Horizon on, cinnabar was used to decorate metal and wooden objects [63,70], and to paint murals at sites on the northern coast of Peru [61]. It was still in use in the later Inca and Colonial periods during the XVI century as identified on wooden *qero* cups [71]. Its application as a body painting pigment by elite *Inca* for certain specific occasions [18;31:112], but also by warriors to look more terrifying [18], demonstrates the significance and exclusivity of this colored material compared to iron oxide, much more commonly used in the Andean region. The importance of cinnabar is related to its provenance and association with the mine in Huancavelica in the central Andes, almost 380km from Lima [62,65,68], which was in operation from the Formative period onwards, and became an important source of the mineral for processing of silver in Potosí during the colonial era [31:111]. Although the existence of other sources has not been ruled out, the relevance of the cinnabar of Huancavelica was such that the extracted pigment was sometimes transported several hundred kilometers for its use in specific and sacred contexts [68,72]. The identification of cinnabar on the Pachacamac Idol therefore also gives a material significance to the paint applied on the wooden statue. The choice of cinnabar was probably associated with its brightness, but we cannot exclude specific knowledge related to its provenance, circulation, and production. In the red color of the Idol we therefore see a material that likely combines symbolic and economic dimensions.

The identification of three pigments on the wooden Idol confirms that the object was originally polychromatic. It may have even included additional painted colors that have not been preserved. Although we do not know its specific significance, documenting the item’s polychromatic nature is important as this aspect has been ignored to date, yet was previously reported by Arturo Jiménez Borja, who mentioned that the upper part of the human faces of the wooden Idol was painted with *Ichima—*the name used for cinnabar by Garcilaso and then by Morúa and Polo de Ondegardo, two colonial chroniclers [18]—after observing that “carved lines of it display traces of dried blood (?)” [43:74]. It is therefore surprising that none of the early chronicles mention the polychromatic aspect of the Idol, especially considering that during the colonial period these materials were known. For example, Acosta [31:111] indicates for red colors that *“*[…] *this minium o vermilion—which they called llimpi—*, *that they prized much for the same effect that Pliny referred to for Romans and Ethiopians*: *to paint or dye their faces and their bodies with it*, *and of their idols”* (S4 Text). Llimpi was indicated for the first time by Bernabé Cobo in colonial times. It corresponds to the other name used for azogue metal [associated with mercury], the same from which Indians obtained the vermillion used for body paints. Polo indicated the use of both the terms *ichma* or *llimpi* for azogue or *bermellon* [18]. Thus, either the color was entirely lacking in significance to those who accompanied Pizarro when they arrived at the ceremonial center, or reference to it was intentionally omitted to justify the association of the Idol with the Devil and validate its destruction [73]. Another explanation could relate to an earlier loss of polychromy. The Idol could have been painted during the Middle Horizon after having been carved and disappearing for a considerable length of time. The similar date obtained on a wood container of the Wari tradition where the cinnabar was also identified (769–887 AD) demonstrates that its use could have been in vogue for specific purposes and contexts [70]. Nevertheless, the polychromy identified on the Pachacamac Idol still remains a unique example to this day.

Our direct C14-AMS dating of the Idol (1289 +/-25 BP; cal. 760–876 AD) can thus be cross-referenced with pigment material and also with iconography associated with the Wari tradition, corroborating its 700 years history. The fact that it was cared for over time despite possible changes in the ceremonial practices at Pachacamac serves to emphasize the significance of the Idol. Our study confirmed that the wooden post was cut and very likely carved by the *Wari* in the 8th to 9^th^ centuries and indicates that a form of worship of this culture would have been introduced and consolidated at Pachacamac during the Middle Horizon. However, we cannot specify whether the Idol was sculpted at the site or if it was imported, for example, from the Ayacucho region or a *Wari* administrative center near the monumental site. The presence of *Wari* architecture, offerings, and tombs at the site, in addition to the affiliation of the wooden Idol with this culture, reiterates the importance it acquired from that point onward [74]. It does not, however, resolve the debate related to the mechanism of the *Wari* presence at Pachacamac: as a sign of *Wari* elite domination and establishment, or as a social and economic exchange or connection between local and *Wari* elites [74–76].

As its prestige increased, Pachacamac was gradually transformed into a leading center of devotion, until the *Inca* made it an official sanctuary and place of pilgrimage [37,38]. The *Inca* imposed sun worship as the principal belief system of *Tawantisuyu* in all regions and localities annexed to the empire, although they also allowed the veneration of local deities. This could explain a reference to different idols that were present on Pizarro’s arrival: “*Through all the streets of this town and its main doors*, *and around this house*, *there are many wooden idols*, *and they adore them as an imitation of their devil”* [31:132] (S5 Text). Another and earlier dating of a fragment of *lúcumo* wood found inside an orifice on the upper part of a clay pedestal at the Painted Temple dated to 770+/-70 BP (PUCP-83) [43], and therefore contemporary to the Pachacamac Idol as we have demonstrated here, provides evidence that other statues were standing in this place. These results also confirmed that different gods were probably worshipped at the Pachacamac monumental complex until the Spanish conquest of this ceremonial center.

The use of polychromy in mural painting is unequivocally attested to in Pre-Hispanic societies from almost 4000 BP [77]. With the mineral pigments identified here, we add a new form of colorful practice in the Andean region, and in particular one associated with a sacred object of worship, the Pachacamac Idol. Color adds specific significance and symbolic dimensions that we cannot understand at present, but the use of cinnabar for example also carries economic implications related to its provenance, as another red pigment available at Pachacamac could have been used in its place [44].

As has been demonstrated for other traditions from Antiquity in the Old World, the use of colors was related to their material properties, their symbolism but also had economic and social connotations. Pigments add a material dimension to cult practices, but in particular for people who know pigments, their provenance, acquisition, and preparation. Color affects the perception and sensory experience of the spectator [78–80] and can contribute to create a specific connection to the object of devotion, with clear implications for the cult professed to the so-called Pachacamac Idol and for the social relationship between the different actors involved in this practice. As indicated by Skousen, pilgrimage “is relational—it is a complex, affective gathering of people, places, things, substances, emotions, beliefs, memories, and more” [81:15], so we can in effect suggest that polychromy could add a material dimension to pilgrimage to the Pachacamac sanctuary. Here, we make this contribution to the relevance of brightness in the Andean religion and ornaments associated to specific elites [82], ultimately demonstrating the importance of polychromy, a material dimension little discussed.

## Conclusion

The unpublished chemical results obtained in this study show an exceptionally colorful palette for a venerated and sacred wooden statue preserved for nearly 700 years, demonstrating the significance of the Idol for those who worshipped it. As with the societies of Old World Antiquity, for which statues and other objects of adoration were almost certainly decorated with colors, the polychromy revealed in the so-called Pachacamac Idol provides evidence of a similar practice and adds a new material dimension for cult and pilgrimage in the Andean region.

## Supporting information

S1 Text Original sentences of chronicles referenced. (DOCX)

S2 Text Original sentences of chronicles referenced. (DOCX)

S3 Text Original sentences of chronicles referenced. (DOCX)

S4 Text Original sentences of chronicles referenced. (DOCX)

S5 Text Original sentences of chronicles referenced. (DOCX)

S1 Fig Base of the wooden post where wood sampling was realized. (PDF)

S2 Fig Reflective light micrographs of wood sample. (PDF)

S1 Table C14-AMS dating for wood from the Pachacamac Idol. (DOCX)
