# Supplementary material for: Unraveling the polychromy and antiquity of the Pachacamac Idol, Pacific coast, Peru
Source: PLoS One. 2020 Jan 15;15(1):e0226244. doi: 10.1371/journal.pone.0226244 (PMC6961831; doi:10.1371/journal.pone.0226244)
Supplement: S1 Text — (DOCX) [file pone.0226244.s001.docx]

**S1 Text.** Original Sentences translated by us: “deshacer la bóveda donde el ídolo estaba y quebrarle delante de todos”.
