# Supplementary material for: Unraveling the polychromy and antiquity of the Pachacamac Idol, Pacific coast, Peru
Source: PLoS One. 2020 Jan 15;15(1):e0226244. doi: 10.1371/journal.pone.0226244 (PMC6961831; doi:10.1371/journal.pone.0226244)
Supplement: S2 Text — (DOCX) [file pone.0226244.s002.docx]

**S2 Text.** Original Sentences translated by us: “El ídolo estaba en una buena casa bien pintada en una sala muy oscura, hidionda y muy cerrada; tienen un ídolo hecho de palo muy sucio y aquél dicen que es su dios el que los cría y sostiene y cría los mantenimientos”. “Averiguóse que el diablo se reviste, y habla con aquellos sus aliados […] A éste tienen por dios, y le hacen muchos sacrificios”.
