# Supplementary material for: Unraveling the polychromy and antiquity of the Pachacamac Idol, Pacific coast, Peru
Source: PLoS One. 2020 Jan 15;15(1):e0226244. doi: 10.1371/journal.pone.0226244 (PMC6961831; doi:10.1371/journal.pone.0226244)
Supplement: S3 Text — (DOCX) [file pone.0226244.s003.docx]

**S3 Text.** Original Sentences translated by us: “[…] una cueva muy pequeña, tosca, sin ninguna labor; y en medio de ella estaba un madero, hincado en la tierra, con una figura de hombre hecha en la cabeza de él, mal tallada y mal formada…”.
