# Supplementary material for: Unraveling the polychromy and antiquity of the Pachacamac Idol, Pacific coast, Peru
Source: PLoS One. 2020 Jan 15;15(1):e0226244. doi: 10.1371/journal.pone.0226244 (PMC6961831; doi:10.1371/journal.pone.0226244)
Supplement: S4 Text — (DOCX) [file pone.0226244.s004.docx]

**S4 Text.** Original Sentences translated by us: “[…] este minio o bermellón —que ellos llaman llimpi—, el cual preciaban mucho para el mismo efecto que Plinio ha referido de los romanos y etíopes: que es para pintarse o teñirse con él los rostros y cuerpos suyos, y de sus ídolos”.
