# Supplementary material for: Unraveling the polychromy and antiquity of the Pachacamac Idol, Pacific coast, Peru
Source: PLoS One. 2020 Jan 15;15(1):e0226244. doi: 10.1371/journal.pone.0226244 (PMC6961831; doi:10.1371/journal.pone.0226244)
Supplement: S5 Text — (DOCX) [file pone.0226244.s005.docx]

**S5 Text.** Original sentence translated by us: “*Por todas las calles deste pueblo y a las puertas principales dél, y a la redonda desta casa, hay muchos idolos de palo, y los adoran a imitación de su diablo”.*
