# Supplementary material for: Unraveling the polychromy and antiquity of the Pachacamac Idol, Pacific coast, Peru
Source: PLoS One. 2020 Jan 15;15(1):e0226244. doi: 10.1371/journal.pone.0226244 (PMC6961831; doi:10.1371/journal.pone.0226244)
Supplement: S1 Fig — (PDF) [file pone.0226244.s006.pdf]

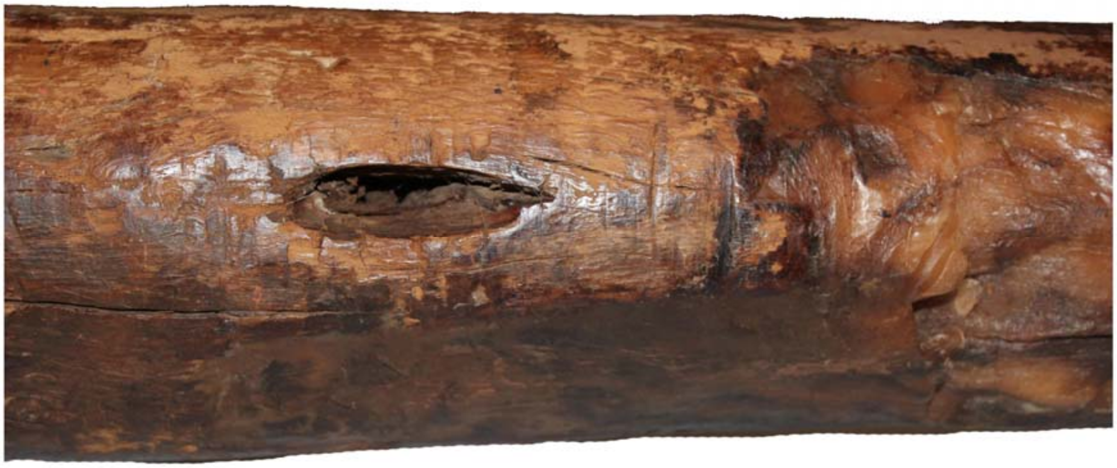

**S1 Fig. Base of the wooden post where wood sampling was realised.**

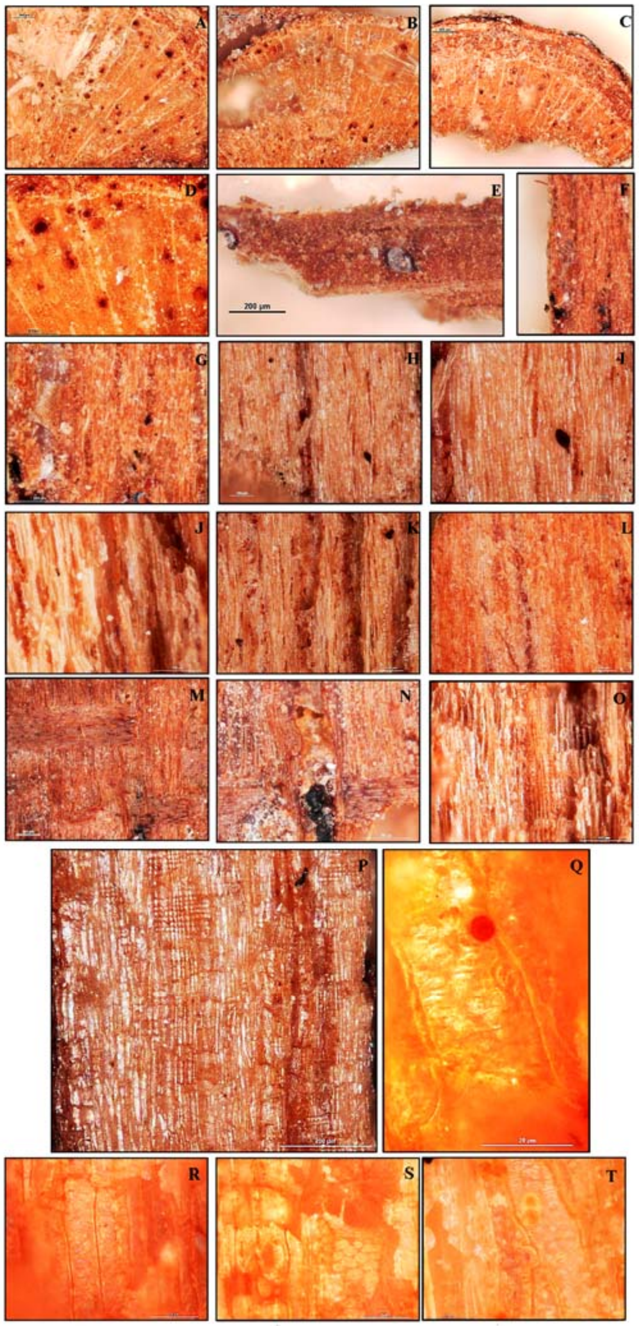

**Fig. S2.** Reflective light micrographs : A-C : juvenile wood transverse section x50; D : juvenile wood transverse section x100; E: mature wood transverse section x100; F : mature wood longitudinal tangential section x50; G : mature wood longitudinal tangential section x100 ; H : juvenile wood longitudinal tangential section x100; I-L : juvenile wood longitudinal tangential section x200; M : mature wood longitudinal radial section x50; N : mature wood longitudinal radial section x100; O : juvenile wood longitudinal radial section x200; P : juvenile wood longitudinal radial section x100; Q-S : vessel pits longitudinal section x1000; T : vessel pits longitudinal tangential section x1000.

**Table S1.** C14-AMS datation for wood of Pachacamac Idol (Results are presented in units of percent modern carbon (pMC) and the uncalibrated radiocarbon age before present (BP). All results have been corrected for isotopic fractionation with an unreported δ13C value measured on the prepared carbon by the accelerator. The pMC reported requires no further correction for fractionation).

| Laboratory code | Sample type | Fraction of modern | | Radiocarbon age | |
|---|---|---|---|---|---|
| | | pMC | 1σ error | BP | 1σ error |
| D-AMS 028819 | wood | 85.18 | 0.27 | 1289 | 25 |
