# Supplementary material for: Unraveling the polychromy and antiquity of the Pachacamac Idol, Pacific coast, Peru
Source: PLoS One. 2020 Jan 15;15(1):e0226244. doi: 10.1371/journal.pone.0226244 (PMC6961831; doi:10.1371/journal.pone.0226244)
Supplement: S2 Fig — (PDF) [file pone.0226244.s007.pdf]

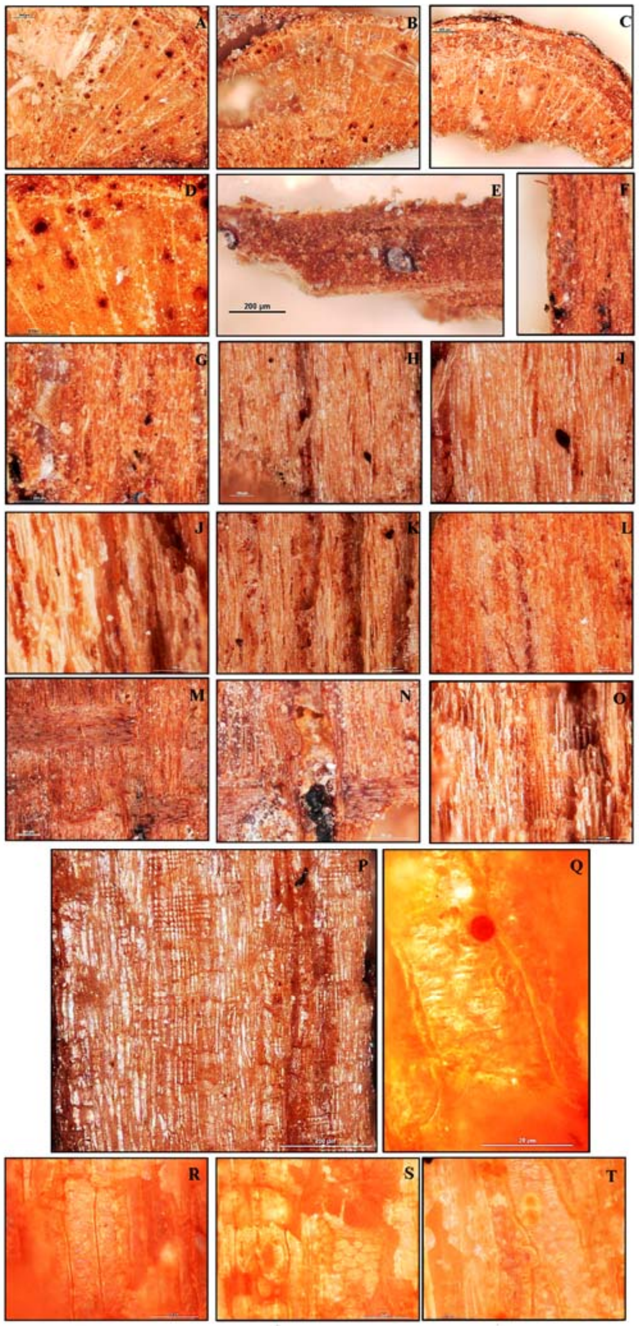

S2 Fig. Reflective light micrographs. A-C : juvenile wood transverse section x50; D : juvenile wood transverse section x100; E: mature wood transverse section x100; F : mature wood longitudinal tangential section x50; G : mature wood longitudinal tangential section x100 ; H : juvenile wood longitudinal tangential section x100; I-L : juvenile wood longitudinal tangential section x200; M : mature wood longitudinal radial section x50; N : mature wood longitudinal radial section x100; O : juvenile wood longitudinal radial section x200; P : juvenile wood longitudinal radial section x100; Q-S : vessel pits longitudinal section x1000; T : vessel pits longitudinal tangential section x1000.
