# Supplementary material for: Unraveling the polychromy and antiquity of the Pachacamac Idol, Pacific coast, Peru
Source: PLoS One. 2020 Jan 15;15(1):e0226244. doi: 10.1371/journal.pone.0226244 (PMC6961831; doi:10.1371/journal.pone.0226244)
Supplement: S1 Table — (DOCX) [file pone.0226244.s008.docx]

**S1 Table. C14-AMS datation for wood of Pachacamac Idol**. Results are presented in units of percent modern carbon (pMC) and the uncalibrated radiocarbon age before present (BP). All results have been corrected for isotopic fractionation with an unreported δ13C value measured on the prepared carbon by the accelerator. The pMC reported requires no further correction for fractionation.

| **Laboratory code** | **Sample type** | **Fraction of modern** | | **Radiocarbon age** | |
| --- | --- | --- | --- | --- | --- |
|  |  | **pMC** | **1**σ **error** | **BP** | **1**σ e**rror** |
| D-AMS 028819 | wood | 85.18 | 0.27 | 1289 | 25 |
